# Combining Denoising Autoencoders and Dynamic Programming for Acoustic Detection and Tracking of Underwater Moving Targets †

**DOI:** 10.3390/s20102945

**Published:** 2020-05-22

**Authors:** Alberto Testolin, Roee Diamant

**Affiliations:** 1 Department of Information Engineering, University of Padova, Via Gradenigo 6/B, 35141 Padova, Italy; 2 Department of General Psychology, University of Padova, Via Venezia 8, 35141 Padova, Italy; 3 Hatter Department of Marine Technologies, University of Haifa, Haifa 3498838, Israel; roee.d@univ.haifa.ac.il

**Keywords:** underwater signal detection, deep learning, Viterbi algorithm, marine monitoring, acoustic detection, SONAR, track before detect

## Abstract

Accurate detection and tracking of moving targets in underwater environments pose significant challenges, because noise in acoustic measurements (e.g., SONAR) makes the signal highly stochastic. In continuous marine monitoring a further challenge is related to the computational complexity of the signal processing pipeline—due to energy constraints, in off-shore monitoring platforms algorithms should operate in real time with limited power consumption. In this paper, we present an innovative method that allows to accurately detect and track underwater moving targets from the reflections of an active acoustic emitter. Our system is based on a computationally- and energy-efficient pre-processing stage carried out using a deep convolutional denoising autoencoder (CDA), whose output is then fed to a probabilistic tracking method based on the Viterbi algorithm. The CDA is trained on a large database of more than 20,000 reflection patterns collected during 50 designated sea experiments. System performance is then evaluated on a controlled dataset, for which ground truth information is known, as well as on recordings collected during different sea experiments. Results show that, compared to the benchmark, our method achieves a favorable trade-off between detection and false alarm rate, as well as improved tracking accuracy.

## 1. Introduction

Underwater detection and localization of moving targets is a key enabling technology for both ecological and security applications. Marine ecology research and well as fishery legislation decisions rely heavily on abundance indications—in this context, the ability to remotely identify marine mammals, pelagic species, or other animals like sea turtles is a game changer in environmental research and management, where abundance is mostly derived from biased, fishery-dependent data [1]. Since acoustic waves propagate well underwater and are known by standards to be safe for marine animals [2], harnessing active SONAR technology that detects targets by acoustic reflections shows great potential to obtain reliable, fishery-independent biomass aggregation during ecological surveys. For example, fish biomass and size spectra can be quantified using directional acoustic methods (echo sounders), which may reflect also perturbation of the entire ecosystem [3]. Active SONAR technology is also already widespread for military applications such as detection of submerged vessels and scuba divers [4]. However, due to the low reflection signature of such targets, the signal-to-clutter ratio (SCR) is usually very small, and progress in the detection of submerged mobile targets through active acoustics is thus still a major challenge.

**Figure 1:**
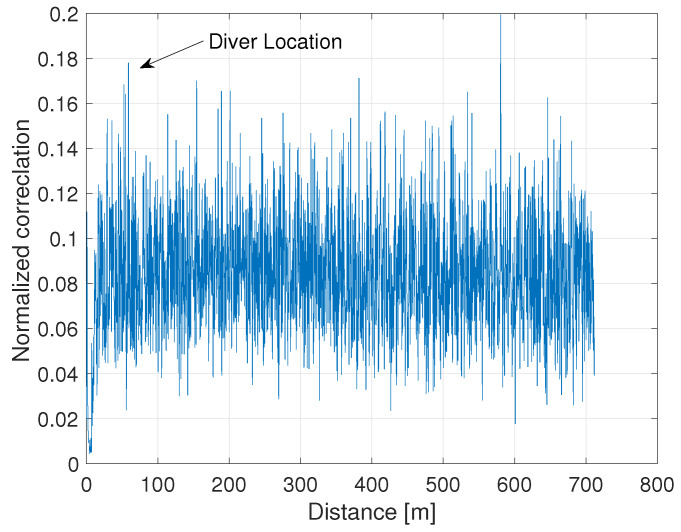
A single reflection pattern from a scuba diver with a low reflection closed-circuit re-breather.

In this paper, we describe a general detection and tracking framework based on a single omni-directional acoustic transceiver, which transmits and receives over a wideband frequency band. This allows flexibility in deployment as well as energy efficiency, such that long-term detection efforts can by made even from small buoys. Analyzing the reflections obtained from the single omni-directional receiver, the main challenge considered here is to detect the target’s-based reflection within the clutter noise. The latter includes stationary reflections (e.g., from rocks or chains) as well as reflections from waves or volume scatters. An example of a reflection response from a scuba diver is shown in Figure 1: note that the diver’s reflection is almost invisible within the clutter.

To detect targets in high clutter, the track-before-detect (TBD) approach has been widely adopted [5]. This approach aims to increase the clutter-to-noise ratio by performing detection over a sequence of observations. The method applies tracking by maximum-likelihood probabilistic data association (ML-PDA) [6], filtering [7], dynamic programming tracking by Markov chain representation [8] and probabilistic multi-hypothesis tracking [9]. Yet, tracking assumes an underline dynamics for the tracked target [10], which may be hard to model for the case of marine animals whose motion tends to be of rapid orientation changes. Considering this, we have recently introduced a probabilistic approach for the case of tracking a single target [11], which allows to detect the target’s reflections within the clutter by using the Viterbi algorithm to identify structured patterns within a time-distance (TD) matrix formed by concatenating matched filter’s outputs sequentially.

However, the main limitation of TBD approaches is the time consuming analysis of sometimes tens of thousands of samples for each reverberation response. Moreover, besides issues of computational complexity the above methods are mostly applied for the detection of a single target, while general solutions should also support efficient detection of multiple targets (see References [12,13] for recent approaches tackling this issue). A promising emerging technology to cope with these limitations is represented by *deep learning* [14], which has recently achieved state-of-the-art performance in a variety of difficult pattern recognition tasks, ranging from image classification [15] to speech recognition [16,17], without requiring domain-specific expert knowledge about the signal characteristics.

In this work we describe a novel method, referred to as Convolutional Denoising Autoencoder–Track Before Detect (CDA-TBD), that combines deep learning with dynamic programming—the former is used as an efficient denoising filter, which removes clutter and highlight target-based reflections in real-time from the time-distance (TD) matrix, while the latter further identifies unique targets and precisely tracks their trajectories. More specifically, we use a convolutional denoising autoencoder (CDA) [18] to highlight potential lines within the TD. Differently from clutter (whose structure is random), such lines likely represent reflections from moving targets. We then apply the forward-backward algorithm, whose states are target’s ID and observations are the values provided by the denoised TD matrix. The latter are considered as emission probabilities, while the state transitions are set by limitations over the motion of the tracked target.

A critical aspect that should be considered for improving the performance of deep learning models is the careful definition of a training data set, which should contain a representative sample of the statistical distribution of the target signals that will be observed during system testing. Since the underwater acoustic reverberation channel is difficult to model analytically, both in terms of the channel impulse response and in terms of the target and clutter reflection patterns, in our work we rely on a large set of real measurements collected from a multitude of more than 50 sea experiments. Each experiment includes both verified clutter and target (fish) reflections, which are systematically combined to create a large-scale training set. To evaluate the generalization capability of the proposed system, we then test it on separate sea experiments carried out with different moving targets (i.e., scuba divers).

To the best of our knowledge, our CDA-TBD approach constitutes the first attempt to combine deep learning with dynamic programming for identifying targets within a reflected acoustic signal. Our contribution is thus threefold:

1. Develop a convolutional denosing autoencoder architecture for the detection of curved lines within a reflection (TD) image.
2. Implement a computationally efficient method that combines deep learning pre-processing with a probabilistic algorithm applied over a track-before-detect approach.
3. Create a statistically large-enough database containing clutter and reflections of acoustic patterns, which we freely share with the community for reproducibility and further research.

Our results show that even in low Signal to Clutter Ratios (SCR), where the reflection pattern from the target is weak, our method yields a favorable trade-off between precision and recall, which exceeds the performance of fully probabilistic approaches (i.e., the Viterbi algorithm) at a much lower computational complexity, and also allows for a more accurate fine-grained tracking of the target path. Further, the results show that our approach easily scales to scenarios featuring multiple targets.

The paper is organized as follows—in Section 2, we discuss the state of the art in probabilistic tracking and ML-based detection. Our system’s model and objectives are outlined in Section 3, along with a description of the sea experiments that allowed to create the large-scale dataset of real measurements used for training and testing our system. Our CDA-TBD methodology is explained in Section 4, and system performance is analyzed in Section 5. Conclusions are drawn in Section 6. Preliminary results, which did not include the TBD method and only explored CDA performance on simulated data, have been recently presented as a conference paper [19].

## 2. Related Work

Detection of targets using active acoustic transmission is nowadays performed by continuous active SONAR (CAS) or by transmission of separated pulses [8]. The former involves multiple narrowband transmissions across the band to detect Doppler components that indicate motion [20]. However, this may induce significant energy consumption and may also harm the bio fauna in the surveyed environment. Further, it may not fit the detection of slowly moving targets. We thus focus on the latter approach. To identify a target within heavy clutter, detection based on a single reflection pattern may fail, and the available literature turns to detection according to a sequence of reverberation patterns. Based on the transmission of consecutive wideband signals of high processing gain such as chirps, single reverberation patterns are analyzed and concatenated to form a TD matrix [21]. This analysis can be performed by a matched filter (MF) [22], a channel equalization such as orthogonal matching pursuit [23], or detectors based on a local estimation of the noise distribution [24]. Once the TD matrix is formed, the detection is performed through tracking.

Tracking over the TD matrix is possible through filtering, for example by exploiting variants of the Kalman filter [10] or using blind tracking to handle non-Gaussian clutter [21]. Alternatively, clutter could be classified using a mixture of distributions [25], such that detection is matched to local clutter patterns within the reflected pattern. A more common approach uses tracking by a particle filter that learns the clutter’s and target’s probabilistic model though statistically sampling the discrete grid of the state-space. This can also be applied for multi-target scenario of active SONAR [7]. Yet, such filtering directs the solution by a most probable grid search and may thus fail to detect targets in high clutter.

For cases of low SCR, the TBD approach has proved useful (a comparison between TBD approaches can be found in References [5,26]). Instead of detection per-transmission or by relying on a motion model, tracking is performed probabilistically. A common TBD approach is maximum-likelihood probabilistic data association (ML-PDA), which applies a likelihood ratio test to parameters observed from the TD matrix [27], and shows good tracking in low SCRs with applications of SONAR detection [6,28]. Alternatively, TBD can use Bayesian tracking, where dynamic programming is applied on a hidden Markov model, as shown in Reference [8] and in our recent work [11]. Another approach is probabilistic multi-hypothesis tracking (PMHT), which tracks possible targets by explicitly separating target and clutter components [29]. The PMHT approach can be extended to combine features such as the intensity distribution of observations [5,30] or the spatial information obtained by arrays of acoustic receivers [31]. This method is also flexible enough to handle fluctuations of the clutter and target distribution within the TD matrix [32] and Reference [33] even offered an indicative metric to determine the conditions in which tracking is feasible. Yet, while TBD approaches often achieve good results, their main disadvantages are the sensitivity to different target’s dynamic types (especially those unknown a-priori) and the high algorithmic complexity, which prevents their application in realistic scenarios (where the TD matrix might contain hundreds of thousands of elements).

These limitations call for the adoption of innovative computational methods. A promising approach is offered by machine learning, which allows to effectively recognize recurring patterns in high-dimensional data by extracting high-order statistical features from a set of training examples. In particular, deep learning methods are particularly effective in pattern recognition tasks where domain knowledge is limited, because they can automatically learn intricate statistical structure from the data by exploiting multiple levels of representation [34]. Furthermore, once trained deep networks are computationally very efficient, since signal processing can be carried out in parallel hardware using basic algebraic operations [35,36].

Deep learning is being widely used in many engineering fields, ranging from compressed sensing [37] and telecommunications [38,39] to fault diagnosis [40] and video surveillance [41]. Deep learning detection methods achieve impressive performance even when the signal is corrupted by high levels of noise [42,43], suggesting it can be successfully applied also in underwater monitoring. Preliminary work exploiting deep learning for the analysis of passive SONAR has been recently proposed [44,45], highlighting the superiority of deep learning over traditional methods based on Mel frequency cepstral coefficients and Hilbert-Huang transform [46].

Considering the surveyed literature, we can identify three main gaps. The first is the applicability of modern deep learning frameworks for the task of efficiently detecting moving targets within a TD matrix. Specifically, we are interested in exploring the denoising performance of stacked autoencoders when the input image contains high levels of environmental noise. Second, the current statistical and probabilistic approaches cannot provide accurate tracking performance in low complexity when the SCR is low and when the target’s characteristics and dynamics are unknown. Third, the application of active SONAR target tracking is currently performed using large arrays, whose deployment is complex and expensive. A much preferred solution would be a single transceiver, preferably of low energy, that can be deployed from small buoys over long periods of time and provide real time detection capability. Confronting these challenges, in the following we present our CDA-TBD approach that combines denoising autoencoders with a TBD solution.

## 3. Problem Formulation

### 3.1. System Model

Our CDA-TBD system comprises a single transceiver emitting a sequence of wideband signals of short duration. In our experiments, we use 20 chirp signals of duration 10 ms and frequency range 7 kHz–17 kHz. The signals are spaced by a 0.7 s guard interval to suppress reflections from previous emissions, which corresponds to detection for distances of roughly 530 m. The transceiver is omni-directional both in transmission and in reception, such that reflections from all directions are received. We make two assumptions on the target: (1) an upper bound, *w*, on the size of the target, and (2) an upper bound, *v*, on the speed of the target relative to the receiver. While the first bound can be set loose since the explored area is large, the second bound should fit well the target’s expected motion to avoid false detection in low signal-to-clutter (SCR) scenarios.

Without prior knowledge of the target’s reflection pattern, we estimate the reflection pattern by the matched filter

(1) $$\mathrm{MF}\left(\tau ,r\left(t\right)\right)=\frac{\int_{0}^{T_{s}}s\left(t\right)r\left(t-\tau \right)dt}{\sqrt{\int_{0}^{T_{s}}s^{2}\left(t\right)dt\;\int_{0}^{T_{s}}r^{2}\left(t-\tau \right)dt}}\;0<\tau <T_{\mathrm{guard}}\;,$$

where $s\left(t\right),0<t<T_{s}$ and $r\left(t\right),0<t<T_{\mathrm{guard}}$ are the transmitted signal and received reflections of duration $T_{s}$ and $T_{\mathrm{guard}}$, respectively. We use a normalized matched filter to provide an initial detection threshold of the direct path based on our previous work [47]. This allows the alignment of each received signal’s reflection, without the need for time-synchronization. The aligned reflections are then stored in a TD matrix representing the time and distance for each reflection.

Being a representative of the time-varying reflection pattern, the TD matrix includes reflections of clutter or either clutter or target. Formally, for the *i*th emission and at distance *j*, entry (i,j) of the TD matrix is

(2) $$\mathrm{TD}\left(i,j\right)=\left\{\begin{array}{cc} \mathrm{MF}(j,\bar{n}\left(i\right)) & \;\mathrm{clutter}\\ \mathrm{MF}(j,\bar{y}\left(i\right)) & \;\mathrm{target} \end{array}\right.\;,$$

where $\bar{n}\left(i\right),\bar{y}\left(i\right)$ are the sampled vector of the clutter and target reflections, respectively, and the ratio $\mathrm{MF}\left(j,\bar{y}\left(i\right)\right)/\mathrm{MF}\left(j,\bar{n}\left(i\right)\right)$ is the SCR. As the expression in Equation (2) hints, a moving target will show as a curved line in the TD matrix, whereas, due to its random nature, clutter will show as random points. We also note that the TD matrix can represent reflections from static targets such as rocks and anchors, which being stationary, will show on the TD matrix as nearly straight lines. In this work we assume these lines are already discarded, for example, by the process described in Reference [11]. Our goal in this work is thus focused on the identification and tracking of curved lines within the TD matrix.

### 3.2. Data Description

Our deep-learning-based solution requires the availability of a large database of TD matrices, annotated with the corresponding ground truth information about the locations of the target reflections (if any). Producing annotated samples in underwater scenarios is challenging. One option to circumvent this issue could be to train the deep network with a simulative model, and perform data augmentation to generate a large set of annotated images (e.g., References [48,49,50]). However, we argue that this approach would fail for the considered task mainly because of two factors: (1) The TD matrix is a representation of a time-varying impulse response of the underwater acoustic reverberation channel. This channel is hard to model, especially due to the highly non-linear reflection pattern within the target’s body, but also even for a simple clutter reflection from the non-homogeneous sea surface. (2) The creation of the TD matrix based on the normalization in Equation (1) is a non-linear operation that is hard to simulate. For example, in the proximity of a strong reflection the normalization factor would decrease the matched filter result, leading to a shadowing effect that highly depends on the SCR. In light of this, we opted for the creation of a database based on a sea campaign of measurements.

**Figure 2:**
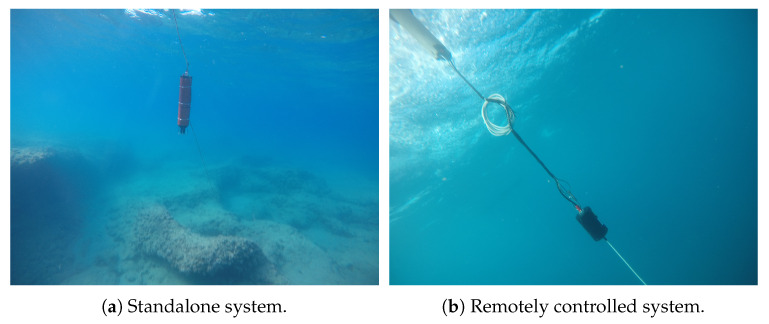
Pictures of the two configurations of transceiver system from two of the sea experiments.

To obtain our database we performed more than 50 sea experiments. Each experiment included a single transceiver deployed from a buoy or a small vessel. As shown in Figure 2, data was obtained from two system configurations: a standalone Subnero M25M acoustic modem, which analyzed the data on the fly emitting signals at frequency range 20 kHz–30 kHz, and a remotely operated EvoLogics LF acoustic modem (S2C-R) that emitted 10 ms duration chip signals at the range of 7 kHz–17 kHz. Both transceivers are fully omnidirectional in the horizontal plane and their response is flat up to 45 degrees in the vertical plane. Since the experiments were performed in shallow area of up to 30 m, this essentially means the emissions and receptions were fully omnidirectional. In the frequency range used, the source level used for both the Subnero and EvoLogics projectors was 180 dB re 1 μPa @ 1 m (the transducer voltage response (TVR) levels are not reported). Balancing the pre-amplifier levels, the nominal receiving sensitivity for both hydrophones was −180 dBV re 1 μPa. In both cases, recording of raw acoustic measurements was done in full duplex, allowing capturing of the direct path. Emissions were done at a period of 0.7 s, allowing reception of targets located up to roughly 530 m (assuming a sound sea velocity of $c_{sound}=1514$ m/s). As such, this is a mono-static acoustic system. In each of the 50 experiments, we recorded at least five hours of data. We then analyzed the recordings offline, in order to identify TD matrices of 20 rows (i.e., 20 reflection patterns) including targets, and TD matrices including clutter-only. The identification was based on the sophisticated procedure discussed in Reference [11], which allows accurate tracking of single targets. This information was sufficient for the aim of offline training the CDA. The experiments were conducted in four different sea environments: (1) at the Red Sea near the shores of Eilat, Israel, at water depth of 30 m and a seabed including a complex reef environment; (2) at the Mediterranean Sea across the shores of Haifa, Israel, at water depth of 15 m with seabed of rocks; (3) at the Mediterranean Sea across the shores of Hedera, Israel, at water depth of 20–10 m with seabed of sand; and (4) at the Mediterranean Sea 11 km west of the northern shores of Israel, at water depth of 160 m with seabed of clay. To verify the reliability of our tagging system, during the experiments we also included targets with verified ground truth information, such as divers dragging buoys with GPS receivers or sharks and tuna fish released after capture for tagging purposes. Further, among others, we identified opportunistic targets such as a dolphin, mackerel, and parrot fish. Our dataset is made freely available through the Open Science Framework (https://osf.io/h79mt/).

**Figure 3:**
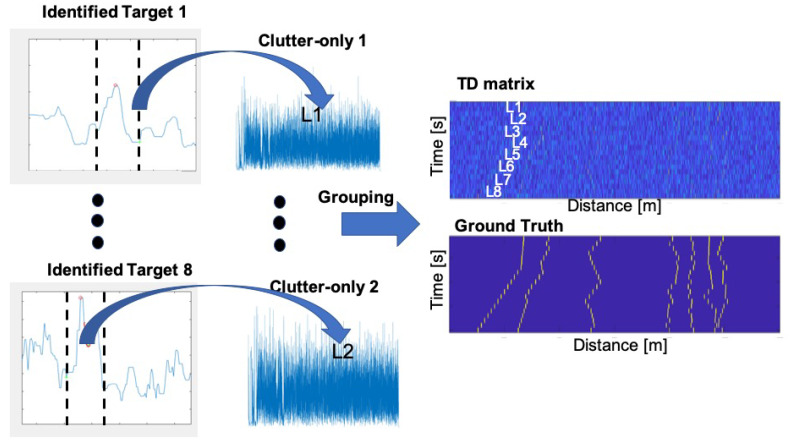
Illustration of the process of data augmentation to create time-distance (TD) matrices. Targets identified by the process described in Reference [11].

Overall, our experiments yielded roughly 1000 different target-based TD matrices, and more than 20,000 clutter-based TD matrices. To balance the database and improve the generalization ability of the CDA, we implemented a data-mixing approach. More specifically, referring to the block diagram in Figure 3, we augmented our database to create a larger number of 10,000 target-based TD metrics by slicing buffers of normalized matched filter outputs around identified target’s reflection, and inserting those over clutter-based TD matrices according to a desired SCR value. Using this methodology, we could also generate different types of reflection lines to reflect various target’s dynamics. This was performed by a smoothed *drunken step* of an auto-regression model of the TD matrix column number where the target is inserted. These locations where then served as the ground truth information for the training and testing of the CDA. The result is a balanced database of 20,000 clutter- and target-based TD matrices: an example of such a formed target-based TD matrix is shown in Figure 3.

## 4. Detection and Tracking Methodology

### 4.1. Convolutional Denoising Autoencoder (CDA)

**Figure 4:**
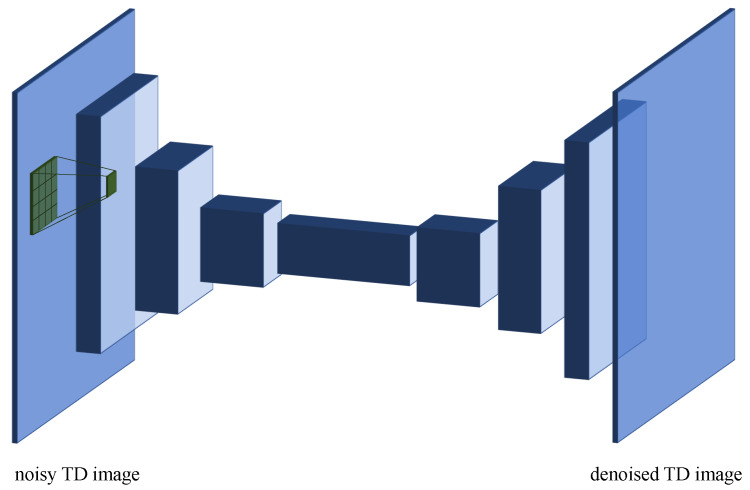
Diagram of the Convolutional Denoising Autoencoder (CDA). The noisy TD image is given as input and processed by a stack of convolutional layers, which detect increasingly more complex features in the signal that are then used by the decoder to produce a denoised TD matrix.

The TD matrix is initially filtered using a deep Convolutional Denoising Autoencoder (CDA), which receives as input the noisy image representing the TD matrix and returns as output a denoised version of the same image (see Figure 4 for a graphical representation). The denoised matrix is then given as input to the TBD algorithm for further processing (see next section).

The autoencoder is composed by four convolutional layers containing, respectively, 24, 48, 72 and 96 kernels of size 4×4, 6×6, 8×8 and 12×16. In order to reduce the dimensionality of the input, the first, second and third layers are followed by pooling layers, with with pool size 1×2 and stride 1×2. Pooling and stride are applied only column-wise because the TD matrix usually contains few rows but tens of thousands of columns. Convolutional (encoding) layers are followed by four deconvolutional (decoding) layers of the same size, which used nearest neighbor as upsampling function. Rectified linear units (ReLUs) are used as activation functions in all layers. A final layer using logistic units is added as a final step to produce output values ranging between zero and one. The CDA architecture and hyperparameters were optimized over a separate validation set using a random search procedure. The CDA was entirely implemented in TensorFlow [51].

To monitor overfitting, the complete data set is split into separate training (60%), validation (20%) and test (20%) sets. The CDA is trained with error backpropagation, using weighted cross-entropy as loss function (the positive class weight was set to 100 in order to counterbalance the sparsity of target detections; extensive simulations showed that the CDA training is robust to variations in this hyperparameter). Learning occurs over mini-batches of 100 images, and continues until the validation loss starts to increase (early stopping).

### 4.2. Detection through Dynamic Programming

In Reference [11], we offered a track-before-detect approach to follow sequences of observations using the Viterbi algorithm constrained to upper bounds on the motion of the target. Specifically, we considered the distance domain of the TD matrix as problem states while the matrix’s rows reflected observations. Each entry of the matrix served as an emission indication, while transition probabilities were chosen by an uniform distribution bounded by the maximum states the target can pass between consecutive observations. While this approach yielded acceptable results also in low SCR, it fits the tracking of only a single target. Further, its computational complexity is extremely high. In this work, building on top of the CDA denoised matrix, we modify the above approach to solve both challenges.

#### 4.2.1. Tracking

**Figure 5:**
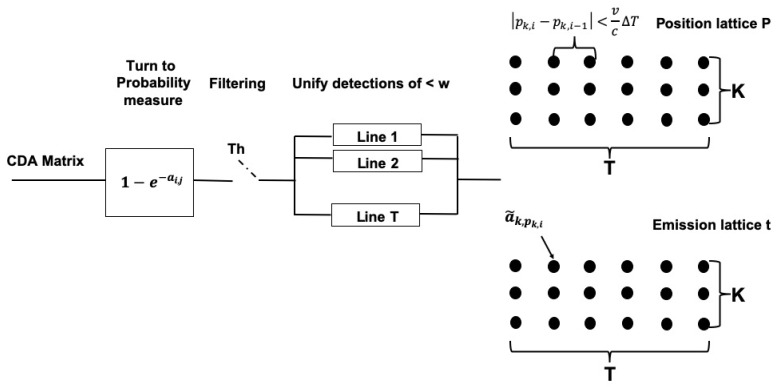
Block diagram for the processing of the CDA matrix before dynamic programming-based tracking.

As illustrated in the block diagram in Figure 5, we start by setting a threshold, Th over the CDA matrix activation output, $a_{i,j}$, for each transmission/row i=0,…,T−1 and each distance slot/column j=0,…,D−1. Setting the sigmoid

(3) $${\bar{a}}_{i,j}=\left\{\begin{array}{cc} 0 & \;a_{i,j}<\mathrm{Th}\\ 1/\left(1+e^{-a_{i,j}}\right) & \;\mathrm{else} \end{array}\right.\;,$$

We transform each matrix entry to a measure of probability. This threshold is determined during the CDA training phase and can be set loose since it lies at the beginning of the detection chain.

Next, considering our upper bound on the size of the target’s reflecting surface, *w*, we identify unique line detections. Specifically, denoting *c* as the sound speed and $F_{s}$ as the sampling frequency, for each row *j*, we unify non-zero entries ${\bar{a}}_{i,j},\;j=0,\ldots ,D-1$ that are spaced less than $w/c\cdot F_{s}$ entries away, to a merged entry whose value is the average of the unified entries while zero forcing its surrounding. The result is a sparse matrix, $\tilde{A}$, of non-zero entries, each reflecting a unique line detection concentrated in one column. On the next step, utilizing our expectation of the target’s maximum speed, *v*, we further compress the smoothed matrix and create a lattice the size of *T* observations and *K* possible targets. These targets are identified by vectors $t_{k},p_{k},\;k=0,\ldots ,K-1$, whose entries $t_{i,k}$ and $p_{i,k}$ contains a non-zero value ${\tilde{a}}_{i,j}$ from $\tilde{A}$ and its location *j*, respectively, such that $p_{i,k}$ is spaced no more than $v/c\cdot \Delta TF_{s}$ from location $p_{i-1,k}$, where ΔT is the guard time between each transmission (in our setting 0.7 s). The result is a merge of the CDA matrix into filtered identified target lines.

Lattice $t_{k}$ as much smaller dimension of K×T compared to the original TD matrix. As a result, we can now apply dynamic programming while still maintaining real-time capability. To that end, we consider the identified targets k=0,…,K−1 as the problem states, the values $t_{i,k},\;i=0,\ldots ,T-1$ as observations, and the transition probability between targets k,q is set by the average of $p_{k}$ and $p_{q}$. Running a dynamic programming like the Viterbi algorithm over the lattice yields the most probable path of a single target. This path reflects the target’s line whose probability entries are the highest and their position in the original TD matrix are the most homogeneous such that the least number of *leaks* to other targets is found. Then, more targets can be found by discarding the already found targets from the lattice and running the dynamic programming again. Finally, to filter detections, assuming the target should exist throughout most of the observation window *T*, we only regard targets *k* whose tracked path by the dynamic programming’s solution is stable throughout at least ρ·T of the lattice, where ρ is a user parameter.

#### 4.2.2. Detection

Once the tracking of several targets is achieved, we turn to make a binary detection regarding the existence of a target. Our detection approach compares the likelihood ratio between the elements of the chosen path to non-identified paths, that is, clutter noise. To that end, for the chosen path $\hat{k}$ and a reference path *j*, we place a threshold, $T_{L}$, over the log-likelihood ratio

(4) $${\mathrm{LLR}}_{j}=\log\left(t_{1,\hat{k}}t_{2,\hat{k}}\ldots \cdot t_{T,\hat{k}}\right)-\log\left({\bar{a}}_{1,j}{\bar{a}}_{j_{2},j}\ldots \cdot {\bar{a}}_{T,j}\right)\;,$$

which compares the likelihood of the chosen path with that of an arbitrary path *j* across the denoised matrix. Then, identifying a set $j=\{j_{1},j_{2},\ldots \}$ of arbitrary paths, none of which belong to lattice $t_{k},\;k=0,\ldots ,K$, we test

(5) $$\mathrm{Detect}=\left\{\begin{array}{cc} 0 & \exists j_{m}\in j:{\mathrm{LLR}}_{j}<T_{L}\\ 1 & \;\mathrm{else} \end{array}\right.\;.$$

### 4.3. Computational Complexity

We consider the emission of *T* consecutive signals whose reflections are recorded to yield a D×T matrix. The complexity of a direct track-before-detect run over this matrix using the Viterbi algorithm is $O(TD^{2})$ (cf. Reference [11]) which, since *D* can be on the order of $10^{4}$ samples, would be very high. Instead, in the CDA-TBD method we propose the denoised TD matrix produces a a lattice of K×T entrees, where *K* is the maximum number of possible targets, which allows to greatly reduce the computational load compared to a pure TBD method. Regarding the CDA pre-processing, the computational cost of a forward pass through a 2D convolution is $O(F_{I}MNmnF_{O})$, where $F_{I}$ and $F_{O}$ are the number of input and output channels, M×N is the size of the feature map, and m×n is the size of the kernel. This bound can be further reduced in the case of deep architectures with square kernels and increasing number of filters [52], as in our case, leading to $O(pF_{I}F_{O})$, where *p* is the largest kernel size.

Overall, the computational complexity of our CDA-TBD method is thus in the order of $O\left(pF_{I}F_{O}\right)+O\left(TK^{2}\right)$, with $p,F_{I},F_{O},K<10^{2}$.

## 5. Results

For the case of single target images, the performance of our CDA-TBD method is validated against two alternative approaches. The first benchmark method, denoted *CDA-Max*, is derived by considering the output provided by the CDA alone. For the tracking task, the target position is estimated by considering the maximum CDA activation at each row: elements should be 1 only in correspondence to the target positions (i.e., the center of the line in the TD image) and 0 elsewhere. For the detection task, in CDA-Max we compare the number of logistic activation along the best path that passes a desired probability $P_{\mathrm{act}}$ to threshold ρ·T. This detection strategy checks that the number of valid reflections along the identified path is significant. Formally, let $\hat{k}$ be the chosen path and $t_{i,\hat{k}},\;i=0,\ldots ,T-1$ its related activation. Then, we set the detection flag

(6) $$\mathrm{Detect}=\left\{\begin{array}{cc} 1 & \exists i=\left\{i_{1},i_{2},\ldots \right\}:t_{i_{j},\hat{k}}>P_{\mathrm{act}},\left|i\right|>\rho \cdot T\\ 0 & \;\mathrm{else} \end{array}\right.\;,$$

which yields a per-TD matrix detection hard decision. The second benchmark method, denoted *Viterbi*, is the “pure TBD strategy” reported in Reference [11], which was shown to outperform other TBD approaches surveyed above. This method compares the emission probability accumulated throughout the chosen path by the Viterbi algorithm to a number of random paths (excluding the chosen path) along the columns of the TD matrix.

As quality metrics, we consider both detection and tracking performance. The former is measured in terms of the receiver operating characteristics (ROC) to explore the trade off between detection and false alarm probability. Tracking error is measured as the average Euclidean distance between predicted position and ground truth. In the following, we show that our CDA-TBD approach clearly outperforms both benchmark solutions. However, we should note that CDA-TBD holds the disadvantage of setting threshold $T_{L}$ by for example, training, whereas, in the CDA-Max approach, both $P_{\mathrm{act}}$ and ρ can be set by some knowledge about the motion and shape of the expected target. This observation emphasizes the need for a sufficiently large dataset, such as the one we share.

Results are first qualitatively shown in terms of representative examples of TD matrices and their denoised version, over which we highlight the target path detected by our CDA-TBD approach. Samples might contain either a single target or multiple targets, and refer to different sea experiments. We then present quantitative measures referring to average errors and ROC curves computed over the entire dataset of more than 20,000 clutter-based TD matrices, separately grouped according to SCR level.

### 5.1. Representative Results and Sea Trial Demonstration

**Figure 6:**
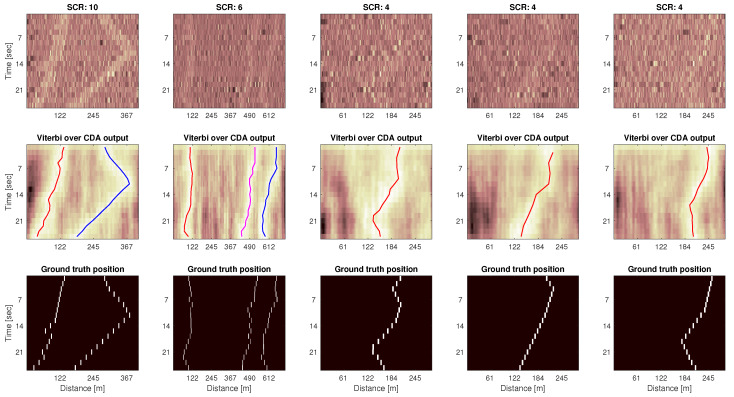
Tracking examples for several TD matrices featuring multiple and single moving targets, at different levels of Signal-to-Clutter ratios. The top row shows the input (noisy) images. The middle row shows the CDA (denoised) images, with the tracked path discovered by our CDA-TBD algorithm superimposed as a red curve. Bottom panels show the corresponding ground truth position of the targets.

A representative set of TD matrices, their denoised version, and the final tracking result is shown in Figure 6 for three different levels of SCR (ground truth target position is reported in the bottom panels), and for cases of a single target and of multiple targets. The leftmost columns demonstrates detection in the case of multiple targets: all target positions are accurately tracked over time. Successive columns show reflections from single targets, where the target’s motion varies between the TD matrices. We observe that in all cases our CDA-TBD method can accurately track the target, even in the presence of very noisy input (e.g., SCR of 4 dB), as indicated by the close match between the tracked line and the ground truth position. Note how the CDA output provides a denoised version of the TD matrix, where the most likely target positions are highlighted. We observe how, after the denoising operation, the position of the target is much better identified than over the original TD matrix.

**Figure 7:**
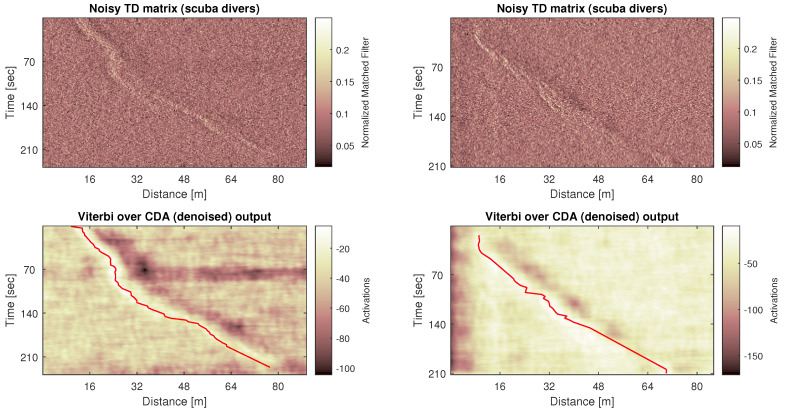
Application of the proposed methodology to TD matrices recorded from the movement of scuba divers.

While the above examples show results for TD matrix created by combining real recordings of clutter and of target’s reflections, our solution should be readily applied also in realistic scenarios where the TD matrix includes both reflection types. Such is the case in Figure 7, where we show the original TD matrix, its denoised version, and the tracking result for two sea experiments including scuba divers. These particular experiments were performed in the Mediterranean Sea, across the shores of Northern Israel, at water depth of roughly 90 m. The sea level was “2” with waves exceeding 0.5 m height. The seabed was a combination of rocks and clay, and the sound speed was roughly steady at 1525±5 m/s at the top 25 m and decreasing linearly to 1510±5 m/s near the bottom. The target were two scuba divers swimming closely. The divers used closed re-breather systems with neoprene-covered air tanks, which made their target strength particularly low. Observing the original TD matrix we note that, while the divers’ path is visible, per acoustic emission, the reflection pattern is very low and compares to the clutter (i.e., low SCR). This motivates the need for pattern-based detection. The denoised matrices shown in Figure 7 emphasize the divers’ path, making it easier to track the target. As clearly observed, the chosen track matches the motion of the target divers.

### 5.2. Statistical Analysis

#### 5.2.1. Detection Performance

**Figure 8:**
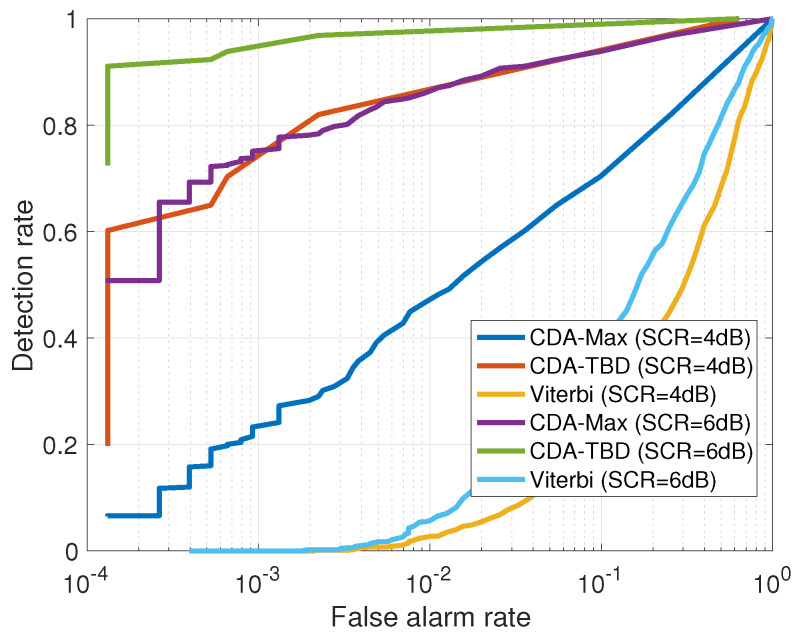
Receiver operating characteristics (ROC) for the three compared methods for SCR = 4 dB and 6 dB. Results shows a favourable trade-off between detection and false alarm rates for CDA-TDB.

Figure 8 shows the ROC performance of all the methods considered, where the different detection and false alarm rates are obtained by changing the detection threshold for each method. We test performance for two relatively low SCR of 4 dB and 6 dB. We observe that, without the denoising step provided by the CDA, performance of the Viterbi algorithm is poor. This is because at low SCR, while the Viterbi approach may catch the right track, the combined probability of the reference tracks are similar to that of the chosen path, thus the likelihood ratio is low. Instead, thanks to the denoising step, in the CDA-TBD case the probability of the reference tracks is low compared to the best path, and the ratio Equation (4) is high even at low SCR. This insensitivity to the clutter noise is also the reason why performance of CDA-TBD is better than CDA-Max. That is, while the latter is making detection decision based on a single denoised observation, the former combines tracks before making a hard decision.

#### 5.2.2. Tracking Performance

**Figure 9:**
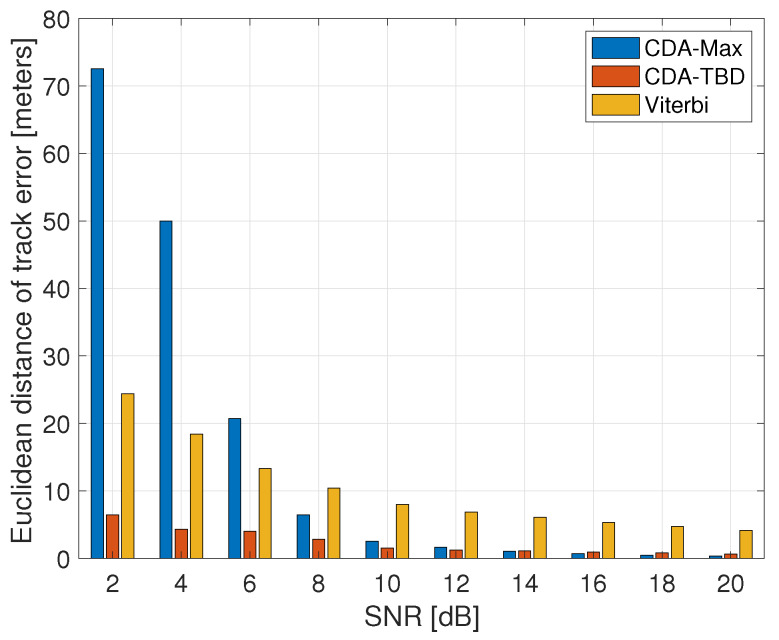
Average tracking error for the three detection approaches as a function of the SCR. Results show resilience of CDA-TBD to high clutter.

Next, we explore the tracking capability of the three approaches. The tracks are obtained by separately setting the detection thresholds for the Viterbi, CDA-Max and CDA-TBD using the ROC curves in Figure 8, according to a desired false alarm rate of $10^{-4}$. Results are shown in Figure 9 as a function of the SCR. We observe that, already at SCR of 6 dB, tracking capability of CDA-Max is low. This happens because taking the maximum value only considers the instantaneous reflection, whereas the other methods observe a global pattern in the denoised matrix. Still, considering a single reflection holds the advantage of independence of the motion of the target. Thus, CDA-Max outperforms the Viterbi approach at high SCR, for which the denoising step is able to filter out much of the clutter. However, the best performance is always given by the CDA-TBD approach, which considers a much lower number of possible targets and it is thus able to produce very accurate results even at low SCR levels. In particular, a sub-meter accuracy is still obtained for low SCR of 8 dB.

## 6. Conclusions

In this paper, we presented an innovative CDA-TBD approach for the efficient detection of multiple mobile submerged targets by active acoustics. Our method takes as input a time-distance (TD) matrix, which concatenates reflections from a train of emitted signals. Motivated by the curved-like pattern created by the target along the time domain, the TD matrix is then filtered through a convolutional denoising autoencoder (CDA) in order to highlight potential patterns in the images. The CDA is trained by an augmented database collected during 50 designated sea experiments, performed under a variety of sea environments. The denoised image is further processed by a probabilistic track-before-detect (TBD) approach to choose paths that fits user-defined expectations about the targets’ maximum size and velocity. This is performed through dynamic programming such that, rather than exploring single reflections, all reflections are considered, thereby allowing detection and tracking even at very low signal-to-clutter ratios. Notably, combining dynamic programming with deep learning allows to cut down computational complexity, which makes the proposed approach a perfect candidate for low-power marine monitoring devices. Moreover, results over the collected dataset of sea experiments show a favourable detection-false alarm trade-off and far better tracking performance over two benchmark schemes. In order to promote further developments, we freely share our dataset with the community.

As a promising research directions, in future work we will explore how detection performance might be improved by training the CDA in a completely unsupervised way, for example by implementing an anomaly detection scheme where a change in the structure of the clutter could be interpreted as the presence of a potential target. Moreover, we plan to implement a real-time version of the proposed method for our SYMBIOSIS monitoring platform, possibly extending the operating range into longer distances.
